# Absence of Clinical and Hemodynamic Consequences due to Posterior Tibial Artery Congenital Aplasia

**DOI:** 10.1155/2015/821094

**Published:** 2015-05-21

**Authors:** Georgios Karaolanis, George Galyfos, Evridiki Karanikola, Viktoria Varvara Palla, Konstantinos Filis

**Affiliations:** ^1^1st Department of Surgery, Vascular Surgery Unit, Laikon General Hospital, Medical School of Athens, Athens, Greece; ^2^Division of Vascular Surgery, 1st Department of Propaedeutic Surgery, University of Athens Medical School, Hippokration General Hospital, Athens, Greece

## Abstract

The exact knowledge of popliteal artery and its branches' anatomic variations is important for the clinical practice of angiology, vascular surgery, and interventional procedures. Congenital absence of the artery leads, in some cases, to early malformations of the extremity in the childhood; however, it may also remain asymptomatic. We present an unusual case of a 76-year-old male patient complaining of paraesthesia in both limbs and bilateral aplasia of posterior tibial artery (PTA). Physical examination, ankle-brachial indexes, before and after exercise, arterial duplex scan, and magnetic resonance arteriography were performed. Arterial pulses for PTA at the level of the ankle were normal; arterial duplex study showed biphasic arterial flow at the level of the ankle. Color duplex ultrasound as well as magnetic resonance arteriography revealed the absence of the PTA in both limbs. The vascularization of the fibula was bilaterally normal. The patient underwent also neurological examination and electromyography, which were normal. The evaluation of the possible clinical signs and symptoms and the hemodynamic consequences of this condition are further discussed.

## 1. Introduction

The vascular system is known to exhibit a wide range of anatomical variations. The presence of vascular anomalies in patients' lower extremities has been documented. Recent studies analyzed these variations using angiography as a method of choice and came to the conclusion that variation in popliteal branching was seen in almost 10% of cases. Absence or aplasia of the posterior tibial artery (PTA) has been rarely described [1]. Compensatory hypertrophy of peroneal artery (PA) associated with hypoplastic or aplastic anterior tibial artery (ATA) or PTA may be a sign of variant arterial supply to the foot [2]. Senior [3] has described the enlargement of PA and the replacement of the PTA in the distal leg and foot through the “peronea magna” and the “great” peroneal artery. When the branches of PA replace the PTA on the ankle, the lower end of it is typically continued into the sole as the lateral plantar artery whereas the medial plantar artery is then usually absent [4].

However, none of the above studies mention the presence of clinical symptoms or signs and hemodynamic consequences as determined in the vascular laboratory and patients' clinical course. We report an unusual case of a male patient who presented with paraesthesia in both legs, with congenital absence of PTA bilaterally but without signs or symptoms of peripheral artery disease.

## 2. Case Report

A 76-year-old male patient was referred to our department complaining of paraesthesia in both legs throughout the last six months. His medical history included aspiration of a kidney cyst 10 years ago and the occurrence of calf vein thrombosis in the right leg 6 years ago (treated with warfarin for 6 months). He had no vascular risk factors (not smoking, healthy nutrition, and regular exercise), no comorbidities (e.g., diabetes mellitus, hypertension, and hyperlipidaemia), and no family history of major cardiovascular events, while he was not receiving any medication.

On examination, palpable pulses of the femoral-popliteal-dorsalis pedis axis and the posterior tibial arteries were present bilaterally, whereas no difference in muscle strength was observed. The ankle-brachial index (ABI) at rest was 1 in both legs. In addition, the levels of toe pressures were normal and the toes-brachial index was >0.7 in both legs. Moreover, treadmill exercise was carried out without significant drop of the ankle pressure and the ABIs. The patient underwent neurological examination and electromyography, which were normal.

**Figure 1:**
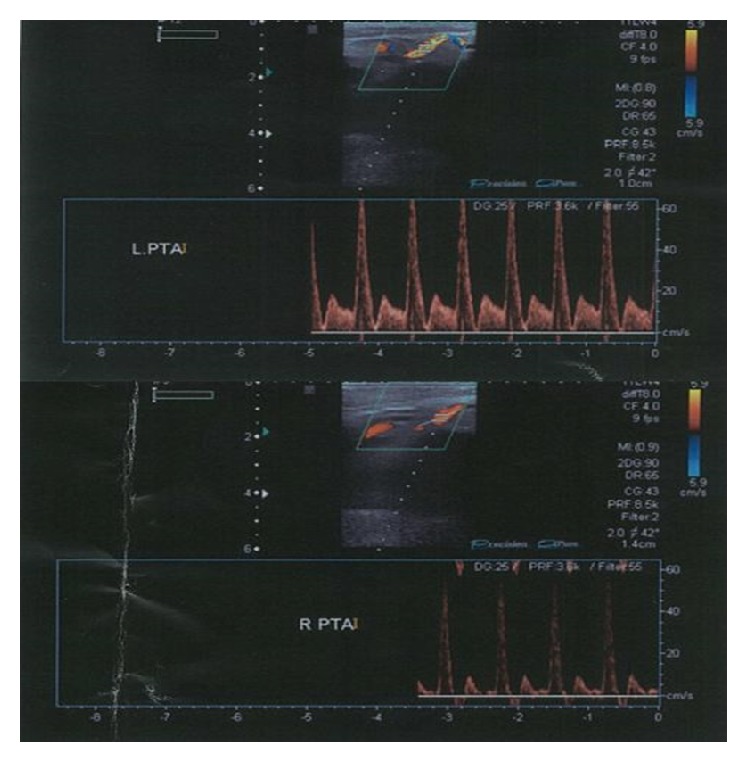
Arterial duplex evaluation shows flow in both posterior tibial arteries.

**Figure 2:**
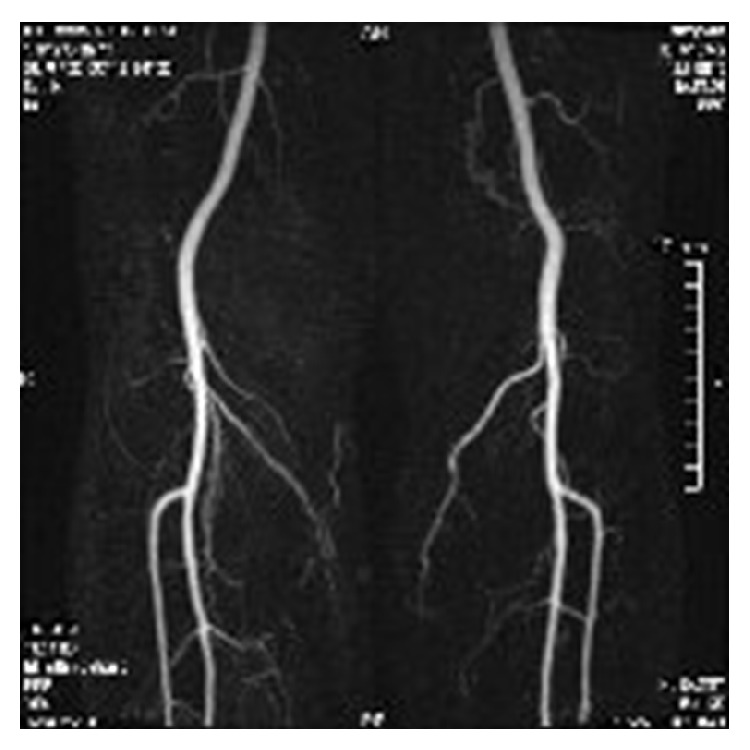
Magnetic resonance arteriography shows the absence of PTA in both limbs through the hypertrophic collateral flow, which takes place in the inferior genicular artery.

Arterial Doppler study (Figure 1) showed biphasic arterial flow of the PTA, at the level of the ankle bilaterally, while color duplex scan showed the congenital absence—from the popliteal artery to the ankle—of PTAs in both limbs. Magnetic resonance arteriography (MRA) (Figure 2) revealed the inferior medial genicular branch, which arises from the superficial femoral artery below the level of the knee and has a very small diameter (approximately 1/4 of the popliteal artery). Moreover, in the same photo, the superior genicular branch just above the knee is also seen. It is noteworthy that the inferior genicular artery is definitely larger than the superior branch in both limbs, resembling a hypertrophic collateral, a finding correlated with the absence of the PTA. In addition, the popliteal artery shows a normal anatomy concerning location, length, and diameter. The anterior tibial artery arises at the normal distance below the knee; the tibioperoneal trunk is in its typical anatomic position; however, the posterior tibial artery, which normally arises 2-3 cm distally, is absent.

Vascularization of the fibula was bilaterally normal through an increased collateral arterial network. More specifically, a recurrent branch of the anterior tibial artery vascularized the proximal epiphysis of the fibula and the peroneal artery vascularized the diaphysis and the distal epiphysis.

The patient underwent follow-up examination every six months for 2 years, including physical examination and arterial duplex studies. Complaints of paraesthesia have been diminished and were definitely not associated with the patient's arterial anatomic variation.

The patient gave informed consent about possible publication of his case. The study was approved by the ethics committee of our hospital.

## 3. Discussion

The knowledge of the popliteal artery variations is important for those performing surgical or percutaneous vascular procedures in the lower leg. Popliteal and peroneal arteries arise from the axial artery whereas the ATA and PTA arise from the femoral system [5]. Stieda supported that the peroneal artery is the main continuation of the popliteal artery and if one of the branches of the popliteal artery is weak or absent, it is then reinforced or replaced by the peroneal artery [6]. The PTA is often the main arterial supply to the foot in patients with clubfoot. Cases with absence of the PTA associated with idiopathic clubfoot in children have been described in the literature as well [7].

In our case, the patient presented without congenital malformation of the lower extremity, while mild paraesthesia was the only symptom. The enlargement of PA according to the ultrasound study and the possible adequate collateral circulation to the fibula represent the main reasons why the patient remained asymptomatic. Vascularization of the fibula remains normal even in the absence of PTA since the nutrient vessel to fibula is provided by the PA [8].

As shown in this case, physical examination alone is not sufficient for the detection of the anatomic variations in the lower extremity arteries. Palpable arterial pulses at the level of the ankle do not exclude congenital absence of even the whole artery. This concurs with studies showing that clinical examination is not independently sufficient to include or exclude a diagnosis of peripheral artery disease [9]. Moreover, normal ABIs and toes' pressures and no symptoms of peripheral artery disease do not exclude PTA absence. Arterial duplex study and magnetic resonance angiography remain as the studies of choice to exclude hypoplastic vessels, total aplasia, or variations in arterial anatomy [10]. If the above noninvasive methods reveal compensatory hypertrophy of PA, this may be a sign of variant arterial supply to the foot and the surgeon or the radiologist has to take this under consideration in case of an operation.

In conclusion, absent tibial arteries may present totally asymptomatic. In addition, normal arterial pulses and normal ABIs do not exclude PTA absence. Therefore, there is a need for detailed preoperative investigation of arterial patency and its normal anatomy before specific types of reconstructive procedures or when endovascular techniques are indicated [8, 9].
